# AFENet: Attention Fusion Enhancement Network for Optic Disc Segmentation of Premature Infants

**DOI:** 10.3389/fnins.2022.836327

**Published:** 2022-04-19

**Authors:** Yuanyuan Peng, Weifang Zhu, Zhongyue Chen, Fei Shi, Meng Wang, Yi Zhou, Lianyu Wang, Yuhe Shen, Daoman Xiang, Feng Chen, Xinjian Chen

**Affiliations:** ^1^Analysis and Visualization Lab, School of Electronics and Information Engineering and Medical Image Processing, Soochow University, Suzhou, China; ^2^Guangzhou Women and Children’s Medical Center, Guangzhou, China; ^3^State Key Laboratory of Radiation Medicine and Protection, Soochow University, Suzhou, China

**Keywords:** optic disc segmentation, multiscale features, attention mechanism, fundus images, premature infants

## Abstract

Retinopathy of prematurity and ischemic brain injury resulting in periventricular white matter damage are the main causes of visual impairment in premature infants. Accurate optic disc (OD) segmentation has important prognostic significance for the auxiliary diagnosis of the above two diseases of premature infants. Because of the complexity and non-uniform illumination and low contrast between background and the target area of the fundus images, the segmentation of OD for infants is challenging and rarely reported in the literature. In this article, to tackle these problems, we propose a novel attention fusion enhancement network (AFENet) for the accurate segmentation of OD in the fundus images of premature infants by fusing adjacent high-level semantic information and multiscale low-level detailed information from different levels based on encoder–decoder network. Specifically, we first design a dual-scale semantic enhancement (DsSE) module between the encoder and the decoder inspired by self-attention mechanism, which can enhance the semantic contextual information for the decoder by reconstructing skip connection. Then, to reduce the semantic gaps between the high-level and low-level features, a multiscale feature fusion (MsFF) module is developed to fuse multiple features of different levels at the top of encoder by using attention mechanism. Finally, the proposed AFENet was evaluated on the fundus images of preterm infants for OD segmentation, which shows that the proposed two modules are both promising. Based on the baseline (Res34UNet), using DsSE or MsFF module alone can increase Dice similarity coefficients by 1.51 and 1.70%, respectively, whereas the integration of the two modules together can increase 2.11%. Compared with other state-of-the-art segmentation methods, the proposed AFENet achieves a high segmentation performance.

## Introduction

Retinopathy of prematurity (ROP) and ischemic brain injury resulting in periventricular white matter (PVWM) damage can lead to visual impairment and even blindness of prematurity (Mcloone et al., 2006; Chen and Smith, 2007). In terms of ROP, retinal vascular proliferative blindness disease frequently affects premature infants with low birth weight. It is reported that 53,000 of the 15 million premature infants worldwide require ROP treatment every year (Agrawal et al., 2021). In addition, periventricular leukomalacia or periventricular hemorrhage may cause PVWM damage, which is considered to be a more common cause of visual morbidity than ROP in premature infants (Mcloone et al., 2006).

As the survival rates of preterm infants in modern neonatal intensive care unit continue to improve, the prevalence of neonatal ischemic brain injury and ROP will also increase. Early diagnosis and timely treatment of ROP and PVWM can effectively reduce visual impairment and prevent disease blindness. The diagnosis and treatment of ROP are based on stage, zone, and plus disease, which reflect the severity of ROP (Aaberg, 1987; International Committee for the Classification of Retinopathy of Prematurity, 2005). For the plus disease, the diagnostic procedure for ROP is to estimate the curvature of blood vessels in a predetermined area around the optic disc (OD), while ROP zoning is defined according to the location of the symptom of ROP relative to the OD. Meanwhile, a previous study has shown that the severity of ROP seems to be positively correlated with a higher proportion of vertical to the horizontal optic diameter (Brodsky and Glasier, 1993). In addition, several studies have reported the association between IVH and optic nerve hypoplasia (Burke et al., 1991; King and Cronin, 1993; Algawi et al., 1995; Oberacher-Velten et al., 2006). Therefore, accuracy OD segmentation of prematurity is extremely significant for the auxiliary diagnosis of these two diseases.

**FIGURE 1:**
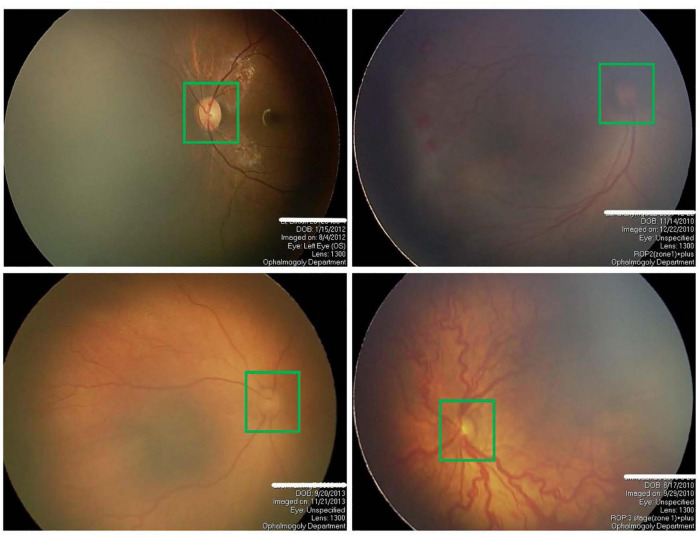
Four examples of fundus images of premature infants, where the optic disc is in the green box.

The segmentation of OD has always been a research hotspot because of its great significance for detecting other anatomical structures in retinal images. OD often appears as bright red circular or oval areas in fundus images, as shown in Figure 1. The irregular OD shape, the diffusion of OD region boundary, and the inconsistency of imaging conditions make OD segmentation very challenging, especially for premature infants. In the past, many related studies on OD segmentation are proposed mainly including traditional algorithms and deep learning (DL) algorithms. Traditional algorithms of OD segmentation use either the intensity of OD region or the point of origination of major vessels for OD localization. The former assumes that the pixel intensity of OD region is higher than that in other parts of the retina (Li and Chutatape, 2001; Walter and Klein, 2001; Chrastek et al., 2002). The main disadvantage of this method is that OD may not be detected correctly in some images because of pathology or uneven illumination. The latter method is based on the assumption that the OD region is the starting point of the major blood vessels of the eye (Hoover and Goldbaum, 2003; Foracchia et al., 2004; Youssif et al., 2007). However, this method may fail when blood vessels are blocked by lesions. With the development of DL technology in recent years, many convolutional neural network (CNN)–based methods, such as fully convolutional network (FCN) (Long et al., 2015), U-Net (Ronneberger et al., 2015), and their variants based networks, have been developed for OD segmentation without considering any prior knowledge, which can overcome the inabilities of traditional technology. For example, Mohan et al. (2018) introduced a prior CNN named P-Net and cascaded the P-Net with their previously proposed Fine-Net, which can further improve the performance of OD segmentation (Mohan et al., 2019). Al-Bander et al. (2018) used a fully convolutional DenseNet with symmetric U-shaped framework for predicting the OD boundary, which achieved a high segmentation accuracy. Fu et al. (2018) proposed M-Net for joint OD and cup segmentation, which is based on multilabel deep network and polar transformation. Liu et al. (2021) proposed a densely connected depth-wise separable convolutional network (DDSC-Net) for joint optic disc and cup segmentation, which outperforms pOSAL (Kadambi et al., 2020), GL-Net (Jiang et al., 2019), M-Net (Fu et al., 2018), and Stack-U-Net (Sevastopolsky et al., 2019). As the encoder and decoder network (U-Net) was proposed for medical image segmentation, many researchers have focused on modifying the U-Net to further enhance the ability of feature learning. For example, context encoder network (CE-Net) was proposed by Gu et al. (2019) for 2D medical image segmentation, which is based on attention mechanism and outperforms the M-Net in the segmentation of OD. Bhatkalkar et al. (2020) modified the basic architectures of DeepLab v3 + and U-Net models (Ronneberger et al., 2015) by integrating an attention module between the encoder and decoder to obtain the better segmentation of OD. Similarly, many variant networks for semantic segmentation tasks have been proposed and achieved high segmentation performance, such as PSPNet (Zhao et al., 2017), Attention U-Net (Oktay et al., 2018), UNet++ (Zhou et al., 2018), and CPFNet (Feng et al., 2020).

In conclusion, U-Net and its variants based on DL hold promise for automated segmentation of OD in digital fundus images. Recently, Agrawal et al. used the original U-Net for the OD segmentation to assist ROP zoning (Agrawal et al., 2021). As far as we know, this is the first time to study the OD segmentation of preterm infants using DL. Different from the segmentation of OD in adults, the OD segmentation of premature infants is more difficult, mainly for the following two reasons: (1) The fundus images of premature infants often have poor image quality and low contrast due to subjective factors such as illumination and eye movement in the actual shooting process of fundus images; (2) due to preterm birth, the retinal structure of preterm infants is often incompletely developed, resulting in low contrast of fundus images, as shown in Figure 1. To handle the above challenges and motivated by the previous successful segmentation networks, we propose a novel attention fusion enhancement network (AFENet) based on the modified encoder and decoder network for automatic segmentation of OD in premature infants. The main contributions of this article can be summarized as follows:

(1) Two novel attention modules including dual-scale semantic enhancement (DsSE) module and multiscale feature fusion (MsFF) module are developed to fuse adjacent high-level semantic information and multiscale low-level detailed information of different levels between the encoder and decoder, respectively.
(2) The proposed DsSE module and MsFF module can be easily integrated in U-shape encoder–decoder network and applied for the OD segmentation of premature infants.
(3) Extensive experiments are conducted to evaluate the effectiveness of the proposed AFENet, and the results show that the proposed AFENet outperforms the state-of-the-art segmentation networks in OD segmentation of premature infants.

The remainder of this article is organized as follows: the proposed method for automatic OD segmentation of premature infants is introduced in section “*Methods*.” Section “*Experiments and Results”* presents the experimental results in detail. In section “*Conclusion and Discussion*,” we conclude this article and suggest future work.

## Methods

### Overview

**FIGURE 2:**
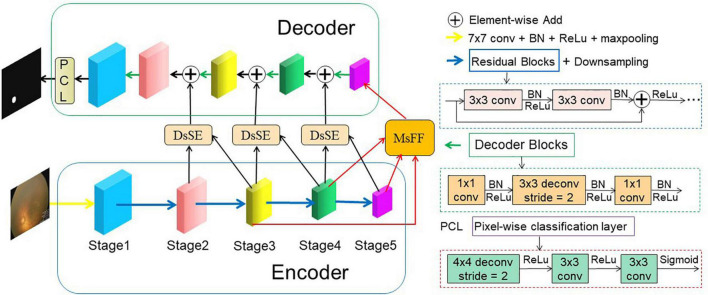
An overview of the proposed AFENet for OD segmentation of premature infants. The AFENet consists of encoder, DsSE module, MsFF module, and decoder, where the encoder and decoder are in blue and green solid boxes, respectively. In addition, “DsSE” and “MsFF” represent the dual-scale semantic enhancement (DsSE) module and multiscale feature fusion (MsFF) module, respectively.

The proposed AFENet for OD segmentation of premature infants is shown in Figure 2, which is based on encoder–decoder U-shape architecture and consists of four main parts: feature encoder, DsSE module, MsFF module, and feature decoder. The feature encoder is used to extract spatial features from the input fundus image, whereas the feature decoder is adopted to construct the segmentation map from the encoded features. The DsSE module is embedded between the encoder and decoder to reconstruct the skip connection, whereas the MsFF module is appended on the top of the encoder to fuse the multiscale feature maps from low-level to high-level features, aiming at reducing the semantic gaps between the high-level and low-level features.

### Feature Encoder

Different from the original U-Net architecture, where each block of encoder consists of two convolutional layers and a max pooling layer for downsampling, the proposed AFENet uses the pretrained ResNet34 (Apostolopoulos et al., 2017) as the backbone of feature extractor, where the global average pooling layer and the fully connected layer are removed. There are two main reasons to use the pretrained ResNet34 as the backbone rather than the original U-Net in the encoding part. First, it is inspired by previous studies (Gu et al., 2019; Feng et al., 2020) that the residual blocks with shortcut mechanism in ResNet can accelerate convergence of the network and avoid gradient vanishing, as shown in the right side of Figure 2. Second, the experimental results in “*Experiments and Results”* also show that compared with the original U-Net, the performance of pretrained ResNet34 as backbone in the encoding part had an overall improvement.

### Dual-Scale Semantic Enhance Module

**FIGURE 3:**
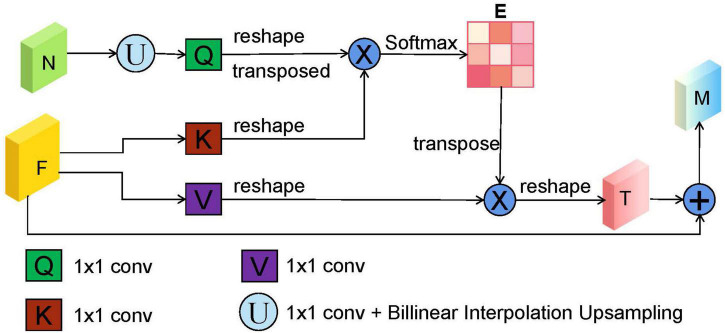
The illustration of dual-scale semantic enhance (DsSE) module. “F,” “N,” “E,” and “M” represent the current input feature map, adjacent next feature map, similarity matrix, and output feature map, respectively. “U” is an upsampling operation, which is obtained by a 1 × 1 convolution and bilinear interpolation upsampling operation. In addition, “Q,” “K,” and “V” are similar to the three branches of self-attention mechanism (query, key, and value), which are realized by three 1 × 1 convolutions.

In the original U-shape network, the skip connection between the encoder and decoder is concatenation operator, which is used to make up for the loss of fine information caused by downsampling. However, it may ignore global information, introduce irrelevant clusters, and produce semantic gap due to the mismatch of receiving domain (Feng et al., 2020). To solve the above problems and highlight salient features, we designed a DsSE module as shown in Figure 3, in which the global semantic information from the next adjacent high-level feature map is fused to enhance the semantic contextual information and reconstruct the skip connection.

In the DsSE module, the skip connection is reconstructed by combining the current feature map with the next adjacent high-level feature map. Suppose that the current input feature map is *F* ∈ ℝ^*C*,*H*,*W*^ and its next adjacent feature map is N∈ℝ^2*C*,*H*/2.*W*/2^. As can be seen from Figure 3, the proposed DsSE module mainly consists of six steps:

(1) To reduce the dimension of weights and computational cost, a 1 × 1 convolution is first used to map the feature map *N* into the same channel space as *F*, and then we upsample the low-dimension feature map to get the same size as *F via* bilinear interpolation, which is denoted as N_*up*_∈ℝ^*C*,*H*,*W*^.

(1) $${\text{N}}_{u\,p}=U\,p\,s\,a\,m\,p\,l\,e\,\left(C\,o\,n\,v\,1\times 1\,\left(N\right)\right)\in {\mathbb{R}}^{C,H,W}$$

(2) Three 1 × 1 convolutions are used to encode the feature map N_*up*_ to query (*Q*) and encode the feature map *F* to key (*K*) and value (*V*), respectively.

(2) $$Q=C\,o\,n\,v\,1\times 1\,\left(N_{u\,p}\right)\in {\mathbb{R}}^{C/r,H,W}$$

(3) $$K=C\,o\,n\,v\,1\times 1\,\left(F\right)\in {\mathbb{R}}^{C/r,H,W}$$

(4) $$V=Conv1\times 1\left(F\right))\in \mathbb{R}{}^{C,H,W}$$

(3) We reshape and transpose Q to *Q*∈ℝ^H*W,*C*/*r*^, and reshape *K* to *K*∈ℝ^*C*/*r*,H*W^ and *V* to *V*∈ℝ^*C*,H*W^, where *C*, *H*, and *W* represent the channel numbers, height, and width of the input feature, and *r* is the compression ratio and is set to 16 in our study.

(5) $$Q=\mathrm{Transpose}\,\left(\mathrm{Reshape}\,\left(Q\right)\right)\in {\mathbb{R}}^{H*W,C/r}$$

(6) $$K=\text{Reshape}\,\left(K\right)\in {\mathbb{R}}^{C/r,H*W}$$

(7) $$V=\text{Reshape}\,\left(V\right)\in {\mathbb{R}}^{C,H*W}$$

(4) We calculate the similarity matrix *E*∈ℝ^H*W,H*W^ between *Q* and *K* to obtain the non-local spatial feature correlation weight guided by global information, as follows:

(8) $$E=\sigma \left(Q*K\right)\in {\mathbb{R}}^{H*W,H*W}$$

where * is the matrix multiplication operation, and σ is Softmax activation function.

(5) The similarity matrix *E* and the corresponding *V* are weighted by matrix multiplication, and we reshape it to obtain the final spatial response *T*∈ℝ^*C*,*H*,*W*^.

(9) $$T=\text{Reshape}\,\left(V*E^{T}\right)\in {\mathbb{R}}^{C,H,W}$$

(6) Finally, we perform element-wise summation operation between *T* and the current input feature map *F* to obtain the final attention output *M*∈ℝ^*C*,*H*,*W*^ as follows:

(10) $$M=F+T\in {\mathbb{R}}^{C,H,W}$$

### Multiscale Feature Fusion Module

**FIGURE 4:**
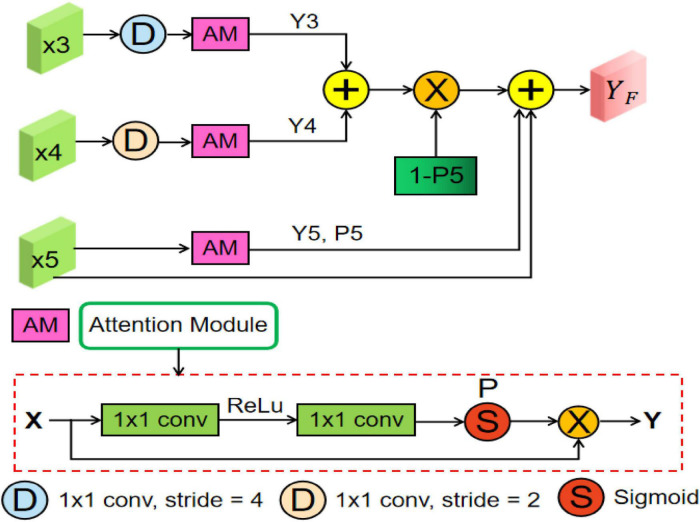
Overview of the proposed multiscale feature fusion (MsFF) module, where “AM” represents an attention module. In addition, “D,” “S,” and “P” are downsampling operation, sigmoid activation function, and confidence map, respectively.

Many previous studies (Oktay et al., 2018; Gu et al., 2019; Feng et al., 2020) have shown that multiscale context information can improve the performance of semantic segmentation, whose core idea is changing global focus to key and local region focus by attention mechanism. However, the above methods may produce the semantic gaps between the low-level and high-level feature maps and ignore the detailed local information. Therefore, to fully utilize the feature interaction between the local context and the global context, an MsFF module is proposed to capture multiscale non-local information with long-range dependency from different levels of encoders, which is illustrated in Figure 4.

As can be seen from Figure 2, the proposed MsFF module is appended on the top of encoder path. Suppose that the feature maps from Stage3, Stage4, and Stage5 are denoted as *X*_*3*_, *X*_*4*_, and *X*_*5*_. As can be observed from Figure 4, the feature maps *X*_*3*_ and *X*_*4*_ are first downsampled to the same size as *X*_*5*_ and then feed them and *X*_*5*_ into the three same attention modules named as AM to generate the corresponding attention feature maps *Y*_*i*_ (*i* = 3, 4, 5) and their corresponding confidence maps *P*_*i*_ (*i* = 3, 4, 5), of which the points with high confidence in *P*_*i*_ (*i* = 3, 4, 5) have a greater possibility to retain the original feature maps values in *X*_*i*_ (*i* = 3, 4, 5), and *vice versa*. As we know, the top feature map *X*_*5*_ of encoder has the stronger abstract semantics and the lower spatial resolution without detailed information in segmentation task. Therefore, the confidence map 1-*P*_5_ represents the lost detailed information in the top feature map *X*_*5*_, where the higher the value of the corresponding position, the richer the detailed information of the corresponding position. In addition, the feature maps *Y*_*3*_ and *Y*_*4*_ with limited semantics and rich detailed information are obtained from relatively shallow layers. Thus, based on the above facts, we can supplement the lost detailed information of the corresponding position on the feature map *X*_*5*_ by using dot product between the confidence map 1-*P*_5_ and the sum of attention feature maps *Y*_*3*_ and *Y*_*4*_. Finally, the feature maps with different scales and semantic information are fused to obtain the final feature maps of the top layer with high-level global feature information and low-level local detailed information, as illustrated in Eq. (11). As illustrated in Figure 4, the AM consists of two 1 × 1 convolutional operations, a ReLU activation function, and a sigmoid activation function. Suppose that the input feature of AM is *X*∈ℝ^*C*,*H*,*W*^. First, the input feature *X* is sequentially fed into a convolutional layer with the kernel size of $\frac{C}{r}\times 1\times 1$, a ReLU activation, a convolutional layer with the kernel size of *C*×1×1, and a sigmoid activation function to obtain the confidence map *P*, where *C* is channel number, and *r* is the compression ratio. Then, we multiply *P* by the input feature *X* to obtain the final output feature map of AM denoted as *Y*∈ℝ^*C*,*H*,*W*^.


(11) $$Y_{F}=X_{5}+Y_{5}+\left(1-P_{5}\right)*\left(Y_{3}+Y_{4}\right)$$

### Feature Decoder

To restore the high-level semantic feature maps generated by the feature encoder and MsFF module, multiple simple decoder blocks are adopted in the decoder path. Previous studies have shown that the deconvolution could learn a self-adaptive mapping to restore the feature maps with more detailed information (Apostolopoulos et al., 2017; Gu et al., 2019). Therefore, the deconvolution is adopted in the feature decoder. As can be observed from Figure 2, the decoder block mainly consists of a 1 × 1 convolution, a 3 × 3 convolution, and a 1 × 1 convolution consecutively. After the last decoder block, the feature map is restored to $\frac{1}{2}$ of the original input image. Finally, we feed it into a 3 × 3 deconvolution and two 1 × 1 convolutions consecutively to obtain the same size segmentation mask as the original input fundus image.

### Loss Function

As illustrated in Figure 2, our framework is an end-to-end DL network, which takes the fundus images as input and outputs the predicted segmentation results. The proposed AFENet is trained to predict each pixel to be foreground or background, which is a pixel-wise classification problem. A main challenge in medical image segmentation is that the segmentation target (OD) takes a small proportion in the fundus images. To solve the class distribution imbalance problem and similar to Bao et al. (2020), Cheng (2020), Feng et al. (2020), Zhu et al. (2020, 2021), Wang et al. (2021), a joint loss *L*_*total*_ is adopted to perform the OD segmentation task, which consists of Dice loss *L*_*Dice*_ and binary cross-entropy loss *L*_*BCE*_. The total loss function combined is defined as follows:


(12) $$L_{t\,o\,t\,a\,l}=L_{D\,i\,c\,e}+L_{B\,C\,E}$$

where,


(13) $$L_{D\,i\,c\,e}=1-\frac{2\,\left|X*Y\right|}{\left|X\right|+\left|Y\right|}$$

(14) $$L_{B\,C\,E}=-\sum_{h,w}\left(1-Y\right)\,\log\left(1-X\right)+Y\,\log\left(X\right)$$

where *X* and *Y* are the segmentation results and the corresponding ground truth, *h* and *w* are the coordinates of the pixel in *X* and *Y*. As can be seen from Eqs (13) and (14), the Dice loss and the binary cross-entropy loss are mainly used to optimize the model in the image and pixel levels, respectively.

## Experiments and Results

### Dataset

In this study, the 1,702 fundus images of premature infants were collected using RetCam3 from Guangzhou Women and Children Medical Center. The gestation ages vary from 26 to 41 weeks, with a mean value of 32 weeks. The collection and analysis of image data were approved by the institutional review board of the Guangzhou Women and Children Medical Center and adhered to the tenets of the Declaration of Helsinki. An information consent was obtained from the guardians of each subject to perform all the imaging procedures. The resolution of the fundus images was 640 × 480. Feng Chen, an ophthalmologist at Guangzhou Women and Children Medical Center, guided the pixel-level annotation. All labeled fundus images were divided into training set, validation set, and testing set, which are shown in Table 1.

**TABLE 1:** Dataset used in this study.

Dataset	Training	Validation	Testing
Num	1,020	341	341

### Experimental Setup

#### Image Processing

To reduce the computational cost and improve the computational efficiency of the model, all the images were resized to 256 × 256 by bilinear interpolation and normalized to (0,1). In addition, online data augmentation, including horizontal flipping, rotations of -10 to 10 degrees and affine transformation, was adopted to prevent overfitting and improve the robust ability of the model.

#### Parameter Setting

The proposed AFE-Net was performed on the public platform Pytorch. We used an NVIDIA RTX3090 GPU with 24-GB memory to train the model with back-propagation algorithm by minimizing the loss function as illustrated in Eq. (12). The Adam was used as the optimizer, where initial learning rate and weight decay were set to 0.0005 and 0.0001, respectively. The batch size and epoch were set to 16 and 100, respectively. To ensure fairness, all the networks in this article were trained with the same optimization schemes, and we saved the best model on validation set in terms of Dice similarity coefficient (Dsc) indicator. The code of the proposed AFENet will be released at https://github.com/yuanyuanpeng0129/AFENet.

#### Evaluation Metrics

To comprehensively and fairly evaluate the segmentation performance of different methods, four evaluation indicators were used, including Dsc and sensitivity (Sen), among which Dsc was the most commonly used metrics in validating the performance of segmentation algorithms (Crum et al., 2006; Milletari et al., 2016; Zhao et al., 2017; Feng et al., 2020). Their definitions are as follows:


(15) $$D\,s\,c=\frac{2\times T\,P}{2\times T\,P+T\,N+F\,P}$$

(16) $$S\,e\,n=\frac{T\,P}{T\,P+F\,N}$$

where TP, TN, FP, and FN are true positive, true negative, false positive, and false negative for pixel classification, respectively.

### Results

#### Qualitative Analysis

**FIGURE 5:**
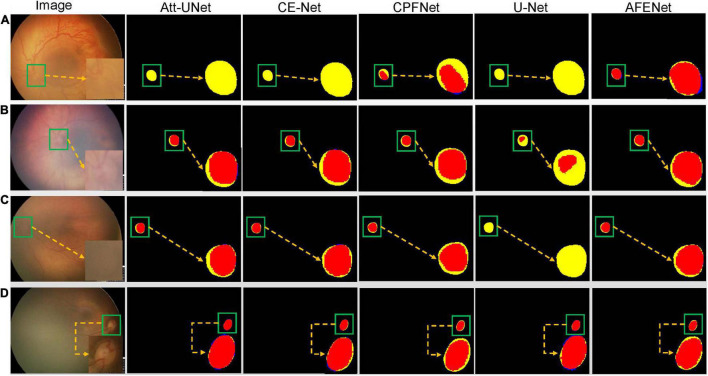
The segmentation results of different methods, where red represents the correctly segmented region, whereas yellow and blue are the results of false-negative segmentation and false-positive segmentation, respectively. To clearly observe the details of the segmentation results, we locally enlarge the segmented target area and place it at the lower right of the corresponding image. In addition, the optic disc is in the green box. **(A)** Represents the fundus image with lesions and blurred optic disc. **(B,C)** Represent normal fundus images with blurred optic disc. **(D)** Represents the normal fundus image with obvious optic disc.

Figure 5 shows four examples of segmentation results of the proposed AFENet and four classical segmentation networks that are widely used in medical image segmentation tasks, where red represents the correctly segmented region, whereas yellow and blue are the results of false-negative segmentation and false-positive segmentation, respectively. Overall, U-Net performs the worst, especially in the case of blurred OD, which has a serious mis-segmentation problem. There are two possible reasons. First, the simple skip connection of concatenation in the original U-Net ignored global information and may introduce interference from local irrelevant features clutter, which led to the poor performance of U-Net in some medical image segmentation tasks with complex pathological features. Second, multiscale context information, which can consider the structure’s surroundings and avoid ambiguous decisions, was not effectively extracted and utilized in each single stage. Compared with U-Net, Att-UNet achieved better segmentation accuracy with few false negatives, which may be due to the introduction of the attention gate (AG) module to guide the model to focus on the salient information in the feature maps of skip connection (Figures 5B,C). Similar phenomena also occurred in CE-Net and CPFNet. The performance of CE-Net and CPFNet was better than UNet, which may benefit from the effective combination of pretrained ResNet34 and global/multiscale context information. However, there are still mis-segmentation problems in the segmentation results of Att-UNet, CE-Net, and CPFNet, especially in Figure 5A. It is worth noting that the proposed AFENet achieved the best segmentation results with fewer false negatives, especially for the segmentation of fundus images with blurred OD, which are common in fundus images of premature infants.

#### Quantitative Analysis

We validated the proposed AFENet on the 341 fundus images of premature infants. For convenience, the basic U-shape model with ResNet34 pretrained on ImageNet as the Baseline method. Table 2 shows the quantitative results of different methods for the OD segmentation. As can be seen from Table 2, DeepLabV3 achieved the worst segmentation performance in terms of all segmentation metrics, which is based on FCN architecture and uses dilated convolution to encode multiscale information at the top of encoder. In addition, DeepLabV3 uses only one-step bilinear interpolation for 16-fold upsampling to upsample the feature map of the encoder to the size of the original image, which lead to insufficient detail information, resulting in poor segmentation performance. Although other methods based on FCN architecture achieved better segmentation performances than DeepLabV3, such as FCN (Long et al., 2015), DANet (Fu et al., 2020), GCN (Peng et al., 2017), and PSPNet (Zhao et al., 2017), the problem of detail information loss caused by downsampling still existed, which may cause the poor performance, especially for the fundus images with blurring OD boundary. In addition, the performance of most U-shape–based networks is better than the networks based on FCN architecture, such as U-Net (Ronneberger et al., 2015), Att-UNet (Oktay et al., 2018), CE-Net (Gu et al., 2019), CPFNet (Feng et al., 2020), CS2Net (Mou et al., 2021), HRSeNet (Wang et al., 2019), DANet (Fu et al., 2020), and TransUNet (Chen et al., 2021), which may be due to the introduction of skip connection between the encoder and the corresponding decoder to alleviate the problem of information loss caused by downsampling. It is worth noting that the proposed AFENet obtains better performance than other segmentation methods in terms of the main evaluation indicator (Dsc). First, compared with Baseline, the performance of the proposed AFENet has been greatly improved, which improves the Dsc and Sen by 2.11 and 1.67% respectively, and achieves 93.12% for Dsc and 93.22% for Sen. Then, compared with other state-of-the-art segmentation networks, the proposed AFENet obtained an overall improvement in terms of the main evaluation indicator Dsc with comparable or less model complexity. For example, compared with the best performance among the comparison methods (CPFNet), the main segmentation evaluation indicator of Dsc of the proposed AFENet increased by 1.88%. Compared with TransUNet (Chen et al., 2021), which has the largest number of model parameters, the performance of the proposed AFENet obtained an overall improvement, especially the Dsc indicator. In addition, compared with GCN (Peng et al., 2017), which has the comparable model complexity, our proposed AFENet has also made great improvement in terms of all evaluation metrics. Especially, we also replaced Backbone with U-Net to further verify the effectiveness and generality of the two modules as shown in Table 2. There are two main findings from Table 2. First, the proposed DsSE module and MsFF module embedded in the U-Net (UNet + DsSE + MsFF) with a small increase in the number of model parameters achieved improvement in terms of the main evaluation indicator Dsc. Second, compared with the UNet + DsSE + MsFF, the proposed AFENet taking the pretrained ResNet34 as Backbone gets an overall improvement in terms of all evaluation indicators (1.99% for Dsc and 0.70% for Sen), which further proves the effectiveness of the pretrained ResNet34 as backbone. These results demonstrate the effectiveness of the proposed AFENet in our task.

**TABLE 2:** The results of comparable experiments and ablation studies on OD segmentation of premature infants.

Methods	Dsc (%)	Sen (%)	Parameters (M)
FCN (Long et al., 2015)	89.42	86.95	18.64
DeepLabV3 (Chen et al., 2017)	85.65	90.07	58.16
DANet (Fu et al., 2020)	89.49	91.26	49.48
GCN (Peng et al., 2017)	88.81	89.53	23.62
PSPNet (Zhao et al., 2017)	91.29	91.68	27.76
U-Net (Ronneberger et al., 2015)	89.87	93.56	7.76
Att-UNet (Oktay et al., 2018)	90.91	**95.48**	8.73
CE-Net (Gu et al., 2019)	91.22	93.74	29.00
CPFNet (Feng et al., 2020)	91.40	89.34	43.27
UNet++ (Zhou et al., 2018)	90.92	92.51	9.16
CS2Net (Mou et al., 2021)	90.64	90.24	8.93
HRSeNet (Wang et al., 2019)	90.88	93.03	**1.63**
UNet + DsSE + MsFF	0.9130	0.9257	8.09
TransUNet (Chen et al., 2021)	0.9012	0.9314	105.28
Baseline	91.20	91.70	21.66
Baseline + DsSE	92.58	92.35	21.93
Baseline + MsFF	92.75	93.34	21.91
AFENet	**93.12**	93.22	22.18

*Bold values are indicate the best performance.*

#### Statistical Significance Assessment

To further investigate the statistical significance of the performance improvement by the proposed AFENet over other state-of-art segmentation networks, paired *t*-test was conducted. The *p*-values of the main evaluation indicator (Dsc) are listed in Table 3. As can be observed from Table 3, all the improvements for Dsc of the proposed AFENet are statistically significant with *p* < 0.05. These results demonstrate that the proposed AFENet can improve the performance of OD segmentation in this study.

**TABLE 3:** Statistical analysis (*p*-value) of the proposed AFE-Net compared with other CNN-based methods.

Methods	Dsc
AFENet-FCN (Long et al., 2015)	< 1E-4
AFENet-DeepLabV3 (Chen et al., 2017)	< 1E-4
AFENet-DANet (Fu et al., 2020)	< 1E-4
AFENet-GCN (Peng et al., 2017)	< 1E-4
AFENet-PSPNet (Zhao et al., 2017)	0.0006
AFENet-U-Net (Ronneberger et al., 2015)	< 1E-4
AFENet-Att-UNet (Oktay et al., 2018)	0.0004
AFENet-CE-Net (Gu et al., 2019)	0.0016
AFENet-CPFNet (Feng et al., 2020)	< 1E-4
AFENet-UNet++ (Zhou et al., 2018)	0.0055
AFENet-CS2Net (Mou et al., 2021)	0.0001
AFENet-HRSeNet (Wang et al., 2019)	< 1E-4
AFENet-UNet + DsSE + MsFF	0.0004
AFENet-TransUNet (Chen et al., 2017)	< 1E-4
AFENet-Baseline	0.0019

### Ablation Experiments

#### Ablation Study for Dual-Scale Semantic Enhancement Module

As can be seen from Figure 2, we proposed a novel dual-scale semantic enhancement (DsSE) module to replace the simple skip connection in the original U-shape network. To prove the effectiveness of the proposed DsSE module, we conducted a series of ablation experiments for OD segmentation of premature infants. As shown in Table 2, the Baseline + DsSE achieved improvement in terms of all evaluation indexes. Compared with the Baseline, the Dsc and Sen of Baseline + DsSE increased from 91.20 and 91.70% to 92.58 and 92.35%, respectively, which benefits from the fact that the DsSE module can help the U-shape network implicitly learn to suppress irrelevant information and highlight salient features useful for a specific task. These results indicate the effectiveness of the proposed DsSE module.

#### Ablation Study for Multiscale Feature Fusion Module

**FIGURE 6:**
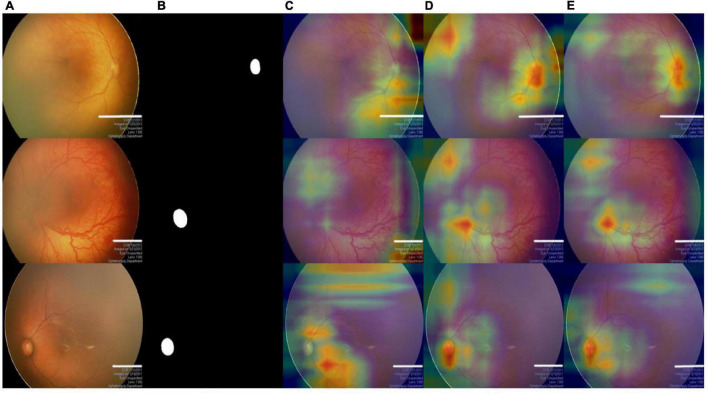
The visualization results of feature maps at the top of the encoder. **(A)** Original images. **(B)** Ground truth. **(C)** Baseline. **(D)** Baseline + MsFF. **(E)** AFENet.

It can be observed from Table 2 that the Baseline + MsFF also obtained an overall improvement in terms of all evaluation indexes. Compared with the Baseline, the Dsc and Sen of the Baseline + MsFF increased by 1.70 and 1.79%, respectively, which benefits the fact that the MsFF module can guide the model to fully utilize the feature interaction between the local context and the global context and promotes the aggregation of low-level weak semantic information with high-level strong semantic information. In addition, to further demonstrate the effectiveness of the proposed MsFF module, the feature maps are visualized for the qualitative analysis, which are the last convolutional outputs of the encoder and show the focus of the network. We compared the visualization results of MsFF-intergraded network (Baseline + MsFF) with Baseline, as shown in Figure 6. It can be seen from Figures 6A–D that the proposed MsFF module can focus on the target object regions better than Baseline, which indicates that the proposed MsFF module can effectively aggregate multiscale context information with long-range dependency and improve the segmentation performance.

## Conclusion and Discussion

Accurate OD segmentation of premature infants is still a challenging task because of the irregular shapes with various sizes, indistinguishable boundaries, and low contrast between background and OD area in fundus images of premature infants. In this study, to solve these problems, a novel segmentation network named AFENet is proposed to segment the OD in fundus images of premature infants. First, to alleviate the feature learning tendency problem that may be caused by the introduction of simple skip connection between the encoder and the corresponding decoder of the U-shaped based networks, a novel attention module named DsSE module is designed to reconstruct the skip connection, which can enhance the semantic contextual information, highlight the salient features, and improve the ability of model learning. Then, to reduce the semantic gaps between the low-level and high-level feature maps, another novel module named MsFF module was developed to fuse multiple-scale feature maps of different levels by using an attention mechanism, which can fully utilize the feature interaction between the local context and the global context and further improve the segmentation performance. Finally, we conducted a series of experiments on the dataset of fundus images of premature infants to verify the effectiveness of the proposed method. Compared with Baseline, the proposed AFENet with two designed attention modules can adaptively focus on target-related area of fundus images and efficiently improve the segmentation performance of OD, which can be seen from Figures 6C,E and Table 2. Compared with other state-of-the-art CNN-based segmentation networks, the segmentation performance of the proposed AFENet has been improved significantly, as shown in Tables 2, 3. As can be seen from Figure 5, compared with the four classical segmentation networks, our AFENet achieves best segmentation results with fewer false negatives and false positives, especially for the segmentation of fundus images with blurred OD, which prove the effectiveness of the proposed AFENet.

The ablation experiments have shown that using the DsSE module or the MsFF module alone can improve the segmentation accuracy, whereas the integration of two modules together can achieve greater improvement. It can be observed from Table 2, for Dsc indicator, that the improvement of 1.51, 1.70, and 2.11% can be achieved by using the DsSE module or the MsFF module alone and the integration of the two modules together, respectively. In addition, taking the proposed MsFF module, for example, the visualization results in Figures 6C,D show that the proposed MSFF module can better focus on the location of key area related to OD segmentation than the Baseline, which further demonstrates that the newly designed MsFF module can accurately learn the effective features.

In conclusion, the proposed AFENet holds promise for OD segmentation in fundus images of premature infants and provides the new opportunities and directions for the zoning of ROP and the auxiliary diagnosis of PVWM damage. We believe that our AFENet can also be applied to other medical image segmentation tasks, which requires further exploration and verification. In the future, we will collect more fundus images of premature infants, aiming at focusing on the further performance evaluation of the proposed method and the possibility of diagnosis for other diseases related to premature infants.

## Data Availability Statement

The in-house dataset (ROP) presented in this article is not readily available because it is constrained by ethics and patient privacy. Requests to access the datasets should be directed to XC, xjchen@suda.edu.cn.

## Ethics Statement

The studies involving human participants were reviewed and approved by the Institutional Review Board of the Guangzhou Women and Children Medical Center and adhered to the tenets of the Declaration of Helsinki. Written informed consent from the participants’ legal guardian/next of kin was not required to participate in this study in accordance with the national legislation and the institutional requirements.

## Author Contributions

YP designed the study, conducted most of experiments, analyzed the experimental results, and drafted the manuscript. WZ and ZC reviewed and revised the manuscript. FS and MW reviewed the manuscript and participated in the design of the experiment. YZ, LW, and YS reviewed the manuscript. DX labeled and interpreted the experimental data in this study. FC collected, labeled and interpreted the experimental data in this study, provided guidance for clinical data analysis, and designed the study. XC designed the study, gave insight in model improvement, reviewed and revised the manuscript. All authors contributed to the article and approved the submitted version.

## Conflict of Interest

The authors declare that the research was conducted in the absence of any commercial or financial relationships that could be construed as a potential conflict of interest.

## Publisher’s Note

All claims expressed in this article are solely those of the authors and do not necessarily represent those of their affiliated organizations, or those of the publisher, the editors and the reviewers. Any product that may be evaluated in this article, or claim that may be made by its manufacturer, is not guaranteed or endorsed by the publisher.
